# Pla2g2a promotes innate Th2-type immunity lymphocytes to increase B1a cells

**DOI:** 10.1038/s41598-022-18876-4

**Published:** 2022-09-01

**Authors:** Susan A. Shinton, Joni Brill-Dashoff, Kyoko Hayakawa

**Affiliations:** Present Address: Fox Chase Cancer Center, 333 Cottman Ave., Philadelphia, PA 19111 USA

**Keywords:** Developmental biology, Immunology, Diseases, Pathogenesis

## Abstract

Newborns require early generation of effective innate immunity as a primary physiological mechanism for survival. The neonatal Lin28^+^Let7^–^ developmental pathway allows increased generation of Th2-type cells and B1a (B-1 B) cells compared to adult cells and long-term maintenance of these initially generated innate cells. For initial B1a cell growth from the neonatal to adult stage, Th2-type IL-5 production from ILC2s and NKT2 cells is important to increase B1a cells. The Th17 increase is dependent on extracellular bacteria, and increased bacteria leads to lower Th2-type generation. Secreted group IIA-phospholipase A2 (sPLA2-IIA) from the Pla2g2a gene can bind to gram-positive bacteria and degrade bacterial membranes, controlling microbiota in the intestine. BALB/c mice are Pla2g2a^+^, and express high numbers of Th2-type cells and B1a cells. C57BL/6 mice are Pla2g2a-deficient and distinct from the SLAM family, and exhibit fewer NKT2 cells and fewer B1a cells from the neonatal to adult stage. We found that loss of Pla2g2a in the BALB/c background decreased IL-5 from Th2-type ILC2s and NKT2s but increased bacterial-reactive NKT17 cells and MAIT cells, and decreased the number of early-generated B1a cells and MZ B cells and the CD4/CD8 T cell ratio. Low IL-5 by decreased Th2-type cells in Pla2g2a loss led to low early-generated B1a cell growth from the neonatal to adult stage. In anti-thymocyte/Thy-1 autoreactive μκ transgenic (ATAμκ Tg) Pla2g2a^+^ BALB/c background C.B17 mice generated NKT2 cells that continuously control CD1d^+^ B1 B cells through old aging and lost CD1d in B1 B cells generating strong B1 ATA B cell leukemia/lymphoma. Pla2g2a-deficient ATAμκTg C57BL/6 mice suppressed the initial B1a cell increase, with low/negative spontaneous leukemia/lymphoma generation. These data confirmed that the presence of Pla2g2a to control bacteria is important to allow the neonatal to adult stage. Pla2g2a promotes innate Th2-type immunity lymphocytes to increase early generated B1a cells.

## Introduction

The generation of unmutated autoreactive-polyreactive CD5^+^ B cells, B1a, occurs at the neonatal stage in mice as an outcome from Arid3a^hi^ immature stage cells in fetal/neonatal liver during Lin28b^+^Let7^-^ B-1 cell development^1,2^. Early-generated B1a cells with certain BCRs are maintained and self-renew through life, providing immediate protection against bacteria and viral infection^3–5^. During aging, some B1a cells reduce CD5 expression, such as by exposure to LPS from gram-negative bacteria^6^. Therefore, these innate-generated B-1-derived B1a origin cells are called B1 B cells (or B-1 B). In old age, some B1 B cells have the ability to generate chronic lymphocytic leukemia (CLL)/lymphoma^6,7^. Aged NZB mice spontaneously generate a higher incidence of B1 lymphoma^8^. In C.B17 mice on a BALB/c background (except C57BL/Ka IgM^b^), spontaneous leukemia/lymphoma generation also occurs, as illustrated by early generation from anti-thymocyte/Thy-1 autoreactive (ATA) BCR B1a cells in ATAμκ transgenic (Tg) mice and anti-non-muscle myosin IIA (aMyIIA) BCR B1a cells in aMyIIA V_H_Q52/D/J knock-in (KI) mice^6,7,9^. Anti-phosphatidylcholine (aPtC) BCR is often found in B1a cells in various mouse lines, and some V_H_11^+^aPtC B1a cells can generate monoclonal B cell lymphocytosis (MBL) in PBLs in old age, which was confirmed in V_H_11/D/J KI.C.B17 mice^10^.

**Figure 1:**
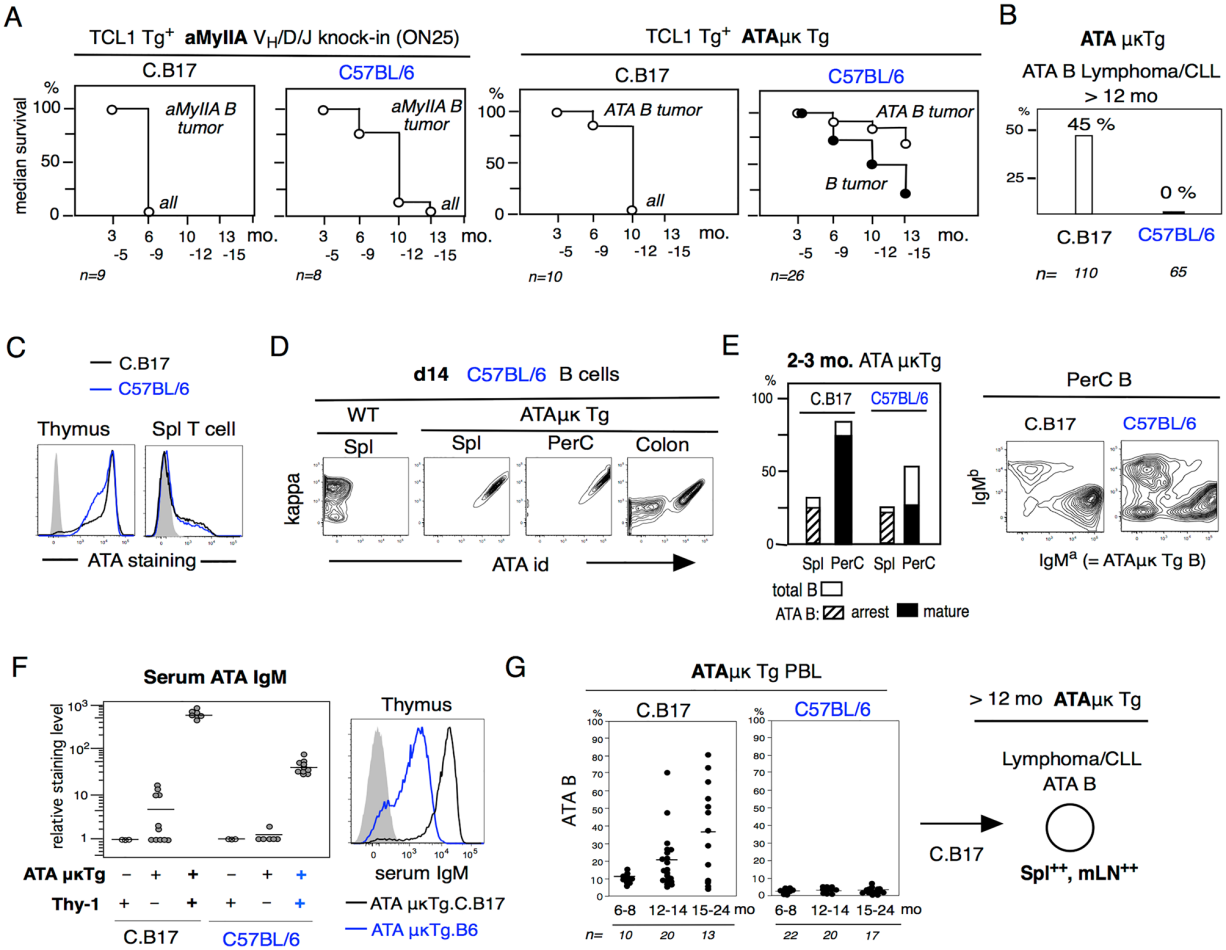
Low and negative B-1 derived lymphoma/CLL generation in C57BL/6 mice.B-1 derived aMyIIA B cells in aMyIIA KI mice and ATA B cells in ATAμκ Tg mice in C.B17 and C57BL/6 backgrounds. (**A**) CLL/lymphoma in aMyIIA KI and ATAμκTg mice crossed with Eμ-hTCL1 Tg mice for the aMyIIA B and ATA B tumor generation stages. (**B**) Spontaneous (TCL1Tg^–^) ATA B lymphoma/CLL generation in > 12 mo mice in the C.B17 background versus negative in C57BL/6 mice. (**C**) ATA IgM (IgM^a+^) staining of T cells in C.B17 and C57BL/6 mice. (**D**) Early generation of ATA B cells in d14 ATAμκTg mice. C57BL/6 mice. (**E**) In the 2–3 mo adult stage, there were fewer total B cells and ATA B cells in the PerC of C57BL/6 mice than in C.B17 mice. In the PerC, the number of non-ATA B cells with endogenous IgM^b+^ B cells was increased in C57BL/6 mice. (**F**) Serum ATA IgM in 2–3 mo C.B17 and C57BL/6 mice crossed with ATAμκTg or ATAμκTg with Thy-1^–^, and ATAμκTg^–^ wild type. Serum ATA IgM from ATAμκTg mice stained with C.B17 thymus. (**G**) Serum ATA B cells in PBLs from the 6 mo to 24 mo stage in C.B17 and C57BL/6 ATAμκTg mice. > 12 mo, generation of ATA B lymphoma (Spl^++^, mLN^++^) in ATAμκTg.C.B17 mice as previously reported^10^.

In ATAμκ Tg mice, C57BL/6 mice exhibit a lower increase in B1a cells from the neonatal to adult stage than C.B17 mice and a lower or negative incidence of leukemia/lymphoma ATA B1 cell generation, as shown in Fig. 1. BALB/c (and C.B17) mice are known to express a unique cyclin-dependent kinase inhibitor p16^INK4a^ allele in the *Cdkn2a* gene, leading to less effective G1 arrest, and NZB mice also express lower levels of the *Cdkn2c* gene encoding p18^INK4c^^11,12^. It is unclear whether this is the only reason for the higher increase in B1a cells and leukemia/lymphoma generation in the BALB/c background, since several additional lymphocyte outcomes also differ between C57BL/6 and BALB/c mice. In C57BL/6 mice, fetal IgAs are lower and microbiota diversity differs in intestine^13^, NKT cells (predominantly invariant NKT, iNKT, with Va14-Ja18 αβT cells in mice) and innate Th2-type ILC2 and NKT2 generation are lower^14–18^, CD4/CD8 T cell ratios are lower^19,20^. In comparison to BALB/c mice, C57BL/6 mice have unique gene differences in contrast to the majority of mouse lines: (1) Mutated Pla2g2a gene leading to deficiency of secreted group IIA-phospholipase A_2_ (sPLA2-IIA) which can degrade phospholipids in bacterial membranes^21,22^. (2) Distinct “signaling lymphocyte activation molecule” (SLAM) family genes differ, leading to lower total NKT and NKT2 generation in C57BL/6 mice compared to the majority of mice^16,23–25^.

Phospholipase A_2_ family proteins (PLA_2_) hydrolyze fatty acids from the *sn-2* position of glycerophospholipids to produce free fatty acids and lysophospholipids^26–28^ through the expression of (1) secreted (s)PLA_2_, which requires Ca^2+^ for catalysis, (2) cytosolic (c)PLA_2_, and (3) Ca^2+^-independent (1)PLA_2_. The sPLA_2_ enzymes comprise the largest PLA_2_ family members, including 11–12 mammalian isoforms that are subdivided into a structural collection of 1/II/V/X subgroups. In the group II sPLA_2_ enzymes, sPLA2-IIA (Pla2g2a gene) has a much higher affinity for phosphatidylethanolamine (PE) and phosphatidylserine (PS), thereby reacting with gram-positive bacteria and with gram-negative bacteria, such as *E-coli* and *Salmonella*. Thus, Pla2g2a has a distinct capacity to degrade bacterial membranes, including the killing of gram-positive bacteria, as a role in host defense against invading bacteria^28–30^. This Pla2g2a was originally found to be secreted from human platelets activated under physiological conditions^31^, and subsequently identified in mammals and chickens as an anti-bacterial^32^. Pla2g2a is widely present in various mammalian tissues, such as the lung, thymus, liver, and kidney, and also high concentrations are found in Paneth cells in intestine, platelets, and tears induced in chronic and acute inflammatory conditions^27,30^. The importance of Pla2g2a in defense from intestinal bacteria was first identified in human colorectal cancers with high frequency of Pla2g2a gene deletion^33^. Distinctly, C57BL/6 mice and Pla2g2a-mutated 129 mice are not able to generate Pla2g2a^21^. These Pla2g2a-deficient C57BL/6 and 129 mice are more strongly susceptible to generating intestinal cancer, including being more sensitive to dextran sulfate sodium (DSS)-stimulated cancer^34,35^. Mucin 2 (Muc2) in goblet cells also plays a role in protecting the intestinal mucosa and maintaining intestinal homeostasis. When Muc 2 is negative, Pla2g2a production in goblet cells can prevent carcinogenesis. In contrast, Muc2 negative C57BL/6 mice that constitutively lack the Pla2g2a role have a higher incidence of generating tumors in the small and large intestine^36,37^.

We considered the potential importance of Pla2g2a in B1 B cell outcomes. Neonatally generated B1a cells are present in both the small and large intestine and mesenteric (m)LNs, together with all B cells from B-1 origin^7,10^. Additionally, B1a cells deposit in the peritoneal cavity (PerC) and lung pleural cavity^3^. IL-5 is important for B1a cell proliferation and self-renewal at this initial stage^38–40^, and IL-5 is produced by Th2-type cells, including ILC2 and NKT2 cells^41,42^. IL-5 and IL-13 production are dependent on IL-25 and/or IL-33^41–43^. IL-25 is secreted from intestinal epithelial tuft cells and from tuft cells in the thymus medulla^44,45^, and IL-33 is a nuclear cytokine that is highly expressed in mouse epithelial barrier tissues and in infected pericryptal fibroblasts^46,47^. Thus, we speculated that changes in microbiota due to the lack of a Pla2g2a role in C57BL/6 mice may have altered IL-25/IL-33 introduction, thus low IL-5 production. We also considered that the difference in microbiota diversity together with the low frequency of NKT2 cells due to SLAM gene differences in C57BL/6 mice may further strongly alter B1a cells at the neonatal-young adult stage and continue to foster the difference throughout life. Our experiments compared Pla2g2a + / + , + /–, –/– mice on a BALB/c background with wild-type C57BL/6 mice, and Pla2g2a + / + , + /–, –/– ATAμκ Tg mice. CD1d knockout mice also used for the importance of increased innate NKT2 cell generation for the initial increase in generated B1 B cells. These data confirmed that the presence of Pla2g2a is important in the neonatal-generated B1a cell increase.

## Results

### Lower and negative B-1-derived lymphoma/CLL generation in C57BL/6 mice

Lack of N-addition at the V-D-J junction in anti-non-muscle MyIIA (aMyIIA) BCR B1a cells generated from B-1 cells in the fetal/neonatal stage was originally found in aMyIIA V_H_/D/J knock-in (KI) C.B17 mice and present in aMyIIA KI C57BL/6 mice. The aMyIIA KI mice crossed with TCL1 Tg^+^ mice on the C57BL/6 background could generate aMyIIA CLL/lymphoma, however, this occurred later than in the C.B17 background (Fig. 1A, left panels). A stronger difference in outcome between C57BL/6 and C.B17 mice were observed in the presence of ATAμκ Tg. These ATA B cells are also of B-1 cell origin. When the TCL1 Tg was crossed into ATAμκ Tg mice, CLL/lymphoma incidence occurred later under the C57BL/6 background than in C.B17 mice, and most that did develop in C57BL/6 mice were non-ATA B cell tumors (Fig. 1A, right). In the absence of the TCL1 Tg, ATAμκ Tg.C.B17 mice showed spontaneously increasing ATA B cells in old aging PBLs, and a high incidence of ATA B cell tumors occurred (45%)^10^. In contrast, such tumors never occurred in ATAμκTg mice. C57BL/6 mice Fig. 1B. aMyIIA KI mice also generated spontaneous lymphoma in the old aged C.B17 background without the TCL1 Tg^7^, but not in C57BL/6 mice.

The ATA IgM generated from ATAμκTg mice binds to a carbohydrate epitope on the Thy-1 glycoprotein, which is strongly expressed on immature CD4^+^ 8^+^ T cells in the thymus, whereas it is expressed at lower levels in mature Thy-1^+^ T cells^9,48^. This Thy-1^+^ ligand for ATA IgM is similarly expressed between C.B17 and C57BL/6 mouse T cells, i.e. There was no autoreactive ligand difference Fig. 1C. At the neonatal stage, ATA B cells are predominant in ATAμκTg mice C57BL/6 mice, including in the colon, consistent with C.B17 mice Fig. 1D ^10^. However, in contrast to C.B17 mice exhibited the continuous presence of B-1-derived mature ATA B cells in the peritoneal cavity (PerC) at the adult stage, and C57BL/6 mice exhibited decreased total B cells in both the spl and PerC (Fig. 1E, left), and decreased ATA B cells in the PerC with increased endogenous B cells (IgM^b^) (Fig. 1E, right). Increased ATA IgMs were present in the serum under the Thy-1^+^ background in both C.B17 and C57BL/6 mice, but these ATA IgM levels were strongly lower in the C57BL/6 background (Fig. 1F, left), and serum IgM binding was lower (Fig. 1F right). These results indicated that initial positive selection of ATA B cells in B-1 development occurred in C57BL/6 mice under the μκ Tg stage, but there was decreased self-renewal by ATA B cells and a change in the BCR repertoire in C57BL/6 mice from the neonate to the initial adult stage. Aged ATAμκTg.CB17 mice had strongly increased ATA B cells in PBL, in contrast to C57BL/6 mice Fig. 1G, and developed lymphoma/CLL ATA B cells that were deposited in the spleen (96%) and mLN (80%), but had a lower incidence in LN or liver, as previously found^10^.

### Reduced IL-5 from Th2-type ILC2s and T cells in Pla2g2a^–/–^ mice

**Figure 2:**
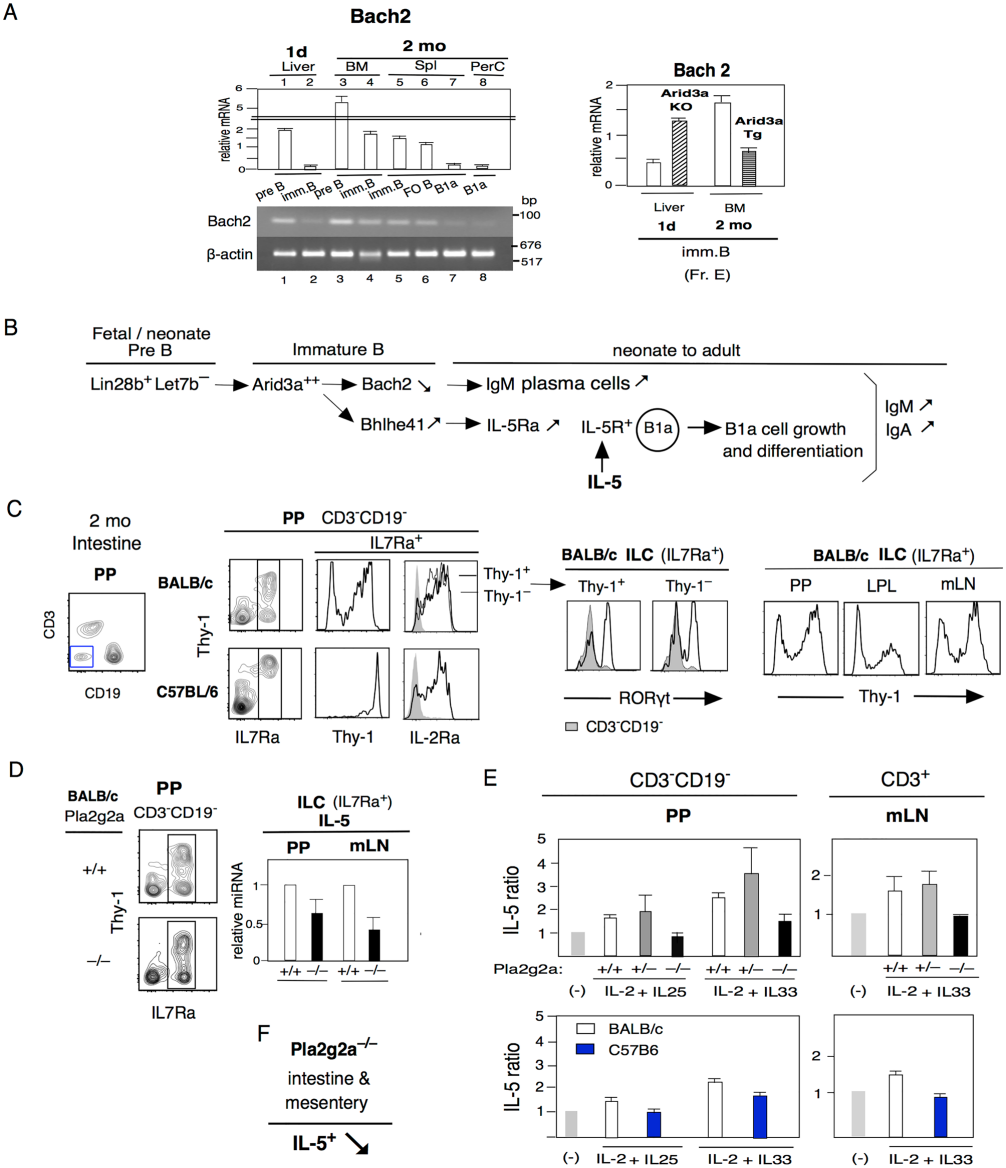
Pla2g2a loss leads to reduced IL-5. Bach2^lo^ allows IgM and IgA secretion and the presence of IL-5 allows IL-5R^+^ B1a cell growth and differentiation, as described in (**B**). (**A**) Analysis of early-generated B1a cell Bach2 levels. Left: Bach2 qRT-PCR of B-lineage 1d liver pre B (Fr.D) and immature B (Fr. E; B220^+^ IgM^+^ AA4.1^**+**^) and 2 mo BM pre B and imm. B with spleen imm. B (AA4^+^), FO B, B1a cells, and PerC B1a cells in C.B17 (n = 5 each; mean ± SE). Bach2 RT-PCR compared with β-actin. Right: Bach2 qRT-PCR in Fr. E in wild type versus Arid3a KO mice in 1d liver, and wild-type versus Arid3a Tg in 2 mo BM (n = 3 each; mean ± SE). (**B**) Arid3a^++^ outcomes are Bach2^low^ and increased Bhlhe41. Bhlhe41^hi^ leads to an IL-5Ra increase in B1a cells, with an IL-5-IL-5R reaction. (**C**) Left: 2 mo intestine ILC (CD3^–^CD19^–^ IL7Ra^+^ ) cell analysis by BALB/c and C57BL/6 mice in PP, with Thy-1, IL-2Ra, and RORγt (ILC3). ILCs were 36% in BALB/c mice and 17% in C57BL/6 mice in the CD3^-^CD9^-^ population. Right: Thy-1^–^ ILCs in PP and LPC in the intestine and in mLNs in BALB/c mice. (**D**) IL-5 mRNA analysis of IL7Ra^+^ ILCs in PPs and mLNs between Pla2g2a + / + and –/– BALB/c mice (n = 3 each; mean ± SE). + / + mice were used as the control. (**E**) IL-5 analysis from PP CD3^-^CD19^-^ cells with IL-2 + IL-25 and IL-2 + IL-33 stimulation in Pla2g2a + / + , + /–. –/– mice (n = 3 each; mean ± SE), and BALB/c versus C57BL/6 mice (n = 2; mean ± SE). mLN CD3^+^ T cells stimulated IL-2 + IL-33 in these mice. Nonstimulated outcome (-) is the control. (**F**) Pa2g2a^–/–^ decreased IL-5 production.

As a Lin28^+^Let7^–^ HSC (hematopoietic stem cell) outcome, pre-B and immature B cell stages are important for B1a cell generation in the fetal/neonatal stage by increased Arid3a^1,2^. These early developed B1a cells strongly secrete IgM and IgA. First, we checked Bach2 in pre-B and immature B cells since promyelocytic leukemia zinc finger (PLZF) generated in neonatal ILCs and T cells leads to Bach2 repression and higher Th2 cell type cytokine generation in T cells^49,50^. We found that Bach2 is also strongly reduced in B-1 B cells at the immature B cell stage. Bach2^low^ in d1 neonatal liver (Fr. E) compared to adult BM, and Bach2 expression was continuously low in mature B1a cells in contrast to adult FO B cells (Fig. 2A, left). Early developed Bach2^low^ B1a cells strongly secrete IgM and IgA Fig. 2B. Loss of Arid3a by Arid3aKO mice increased Bach2 expression in 1d liver, and increased expression of Arid3a by Arid3a Tg decreased Bach2 expression in adult BM at immature Fr. E stage (Fig. 2A, right) (as listed in Supplemental experimental procedure). This confirmed that the low Bach2 expression in immature B cells was generated in Lin28^+^Let7^–^Arid3a^hi^ cells.

After initial B1a cell generation, B1a cell growth occurs. IL-5 allows an increase in B1a cells and increases IgM and IgA production by promoting differentiation toward IgM plasma cells, and the loss of IL-5 or IL-5 receptor (IL-5R) reduces B1a cells^38–40^. High Arid3a expression leads to increased Bhlhe41, and Bhlhe41 promotes IL-5R expression^1,51^Fig. 2B. The IL-5R^+^ B1a cells continued to express the highest IL-5R-expressing B cells in the adult stage. Thus, the IL-5 level is important. Original neonatal B1a cells from B-1 development are present in the intestine-mLN, including in Peyer patches (PP) and colon as found in ATA, aMyIIA, and aPtC B1a cells, and B1a cells deposited to PerC^7,10^. To assess whether loss of Pla2g2a in the BALB/c background leads to changes in IL-5, we generated BALB/C mice. Pla2g2a + / + , + /–, and –/– mice and analyzed PP and mLN. Initial strong IL-5 generation in intestine-mLN is generated from IL-25- or IL-33-stimulated ILC2 cells, and ILC2 cells support self-renewal of B1 cells^52^. CD3^-^CD19^-^ ILC cells, including ILC2, are IL-7Ra^+^^53^ and are also present in intestinal PP Fig. 2C. In contrast to ILCs in C57BL/6 mice that are dominantly Thy-1^high^, BALB/c mice exhibited Thy-1^+^ and Thy-1^low/–^ IL-7Ra^+^ ILCs that are IL-2Ra^+^ in PP, LPL (lamina propria lymphocytes) and mLN Fig. 2C. It is known that ILC2s stimulated with IL-25 can decrease Thy-1 expression, and GATA3 expression in ILC2s is also a negative regulator of Thy-1 expression^54–56^. Furthermore, it is known that the frequencies of ILC2 cells are lower in C57BL/6 mice than in BALB/c mice, including in the lung^14,17,18^. IL-13-producing ILC2 cells can also start to express IL-17 as RORγt^+^ ILC3 cells change upon stimulation^55^, and we observed that RORγt^+^ (ILC3) cells are also Thy-1^–^ ILCs, although they are lower (Fig. 2C, right).

As shown in Fig. 2D, IL-7Ra^+^ ILCs in BALB/c.Pla2g2a^-/-^ mice had a similar fraction of Thy-1^–^ cells in PP (also in mLN) as Pla2g2a^+/+^ mice. However, the IL-5 mRNA level was lower in Pla2g2a^-/-^ mice than Pla2g2a^+/+^ mice (Fig. 2D right), indicating a lower number of ILC2 cells. Since the multipotent progenitor cell type 2 (MPP^type2^; CD3^-^CD19^-^ IL-7Ra^–^ Thy-1^–^ and IL-33 receptor ST2^–^) cells can also be stimulated with IL-25 to secrete IL-5 and are present in PPs and mLNs^57^ similar to ILC2s, we stimulated total CD3^-^CD19^-^ cells (including ILCs and MPP^type2^) and CD3^+^ T cells from PPs and mLNs with IL-25 or IL-33 together with IL-2, since IL-2 is known to further potentiate IL-5 generation^52^. CD3^-^CD19^-^ cells in PP showed low IL-5 in Pla2g2a^-/-^ mice, which is similar to C57BL/6 mice (Fig. 2E left), and CD3^-^CD19^-^ cells in mLN also often had lower IL-5 generation in Pla2g2a^-/-^ mice. Although CD3^+^ T cells in PPs did not show secretion of IL-5, CD3^+^ T cells in mLNs generated IL-5 upon IL-2 + IL-33 stimulation in Pla2g2a^+^ mice in contrast to the reduced IL-5 production in Pla2g2a^-/-^ mice which was also seen in C57BL/6 mice (Fig. 2E right). Thus, IL-5-producing cells were reduced in Pla2g2a^-/-^ mice. Since we confirmed that the levels of the IL-5 receptor, IL-5Ra, on B1a cells were not changed in Pla2g2a + / + , –/– and C57BL/6 mice, we conclude that low levels of IL-5 production explain the phenotype of Pla2g2a^-/-^ mice Fig. 2F.

### Loss of Pla2g2a leads to changes in B1a, MZ B, and the CD4/CD8 ratio

**Figure 3:**
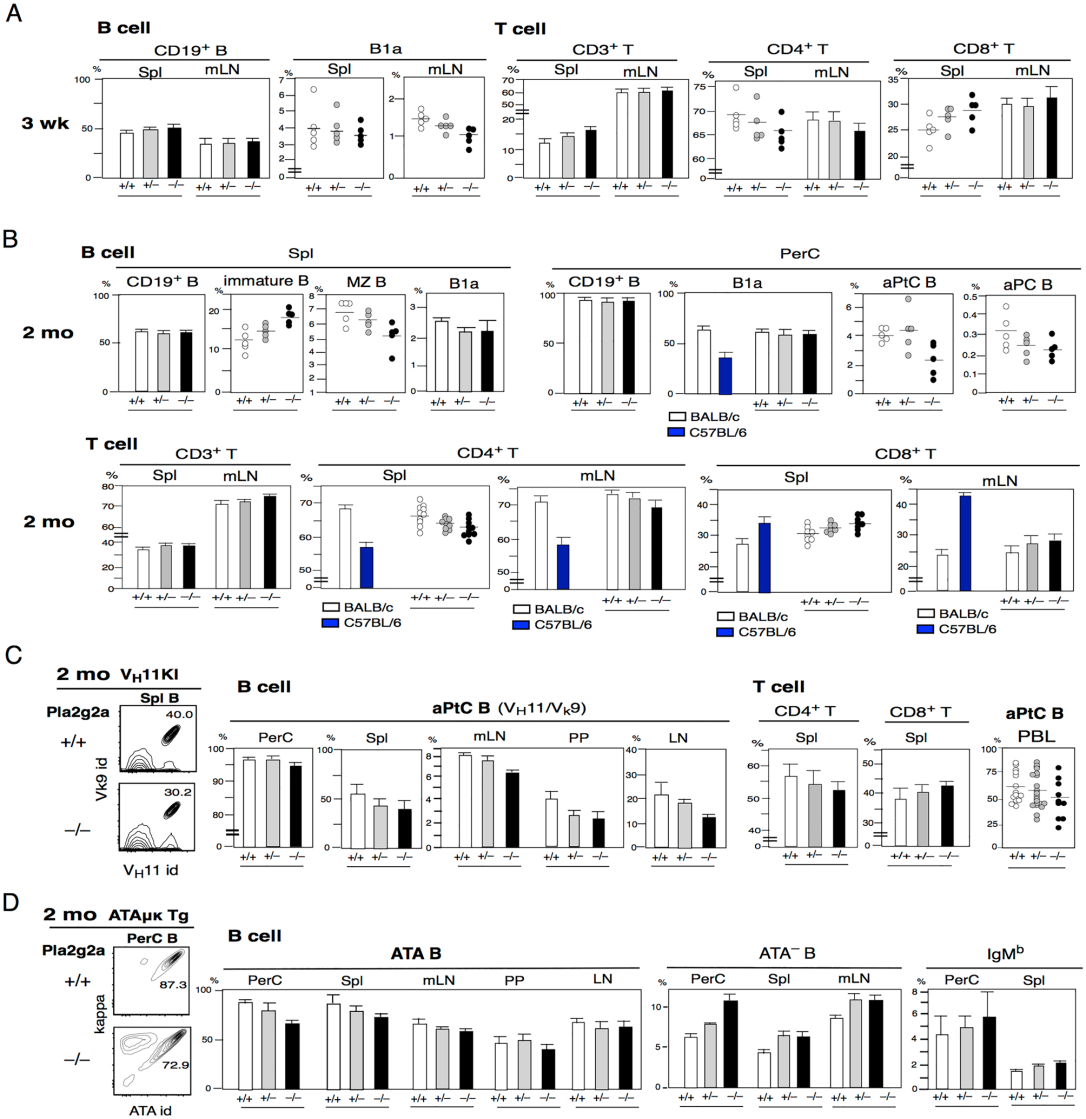
Pla2g2a loss alters B1a, MZ B, and the CD4/CD8 T cell ratio. BALB/c, V_H_11KI, and ATAμκTg mice under Pla2g2a + / + , + /–, –/– backgrounds were subjected to analysis for B and T cells. The majority of data showed the mean ± SE, and some showed individual data with mean percentages and + / + vs –/– P-values as calculated by t-test. (**A**) B (CD19^+^) and T (CD3^+^) cell analysis of 3 wk Pla2g2a + / + , + /–. –/– mice (n = 5 each). For + / + vs –/–: Spl B1a; *P* = 0.429, mLN B1a; *P* = 0.035. Spl CD4^+^ T; *P* = 0.0015, Spl CD8^+^ T; *P* = 0.0002. (**B**) B and T cell analysis of 2 mo Pla2g2a + / + , + /–, –/– mice and wild-type BALB/c and C57BL/6 mice. For B cell spleen and PerC analysis (n = 5 each). For + / + vs –/–: Spl: immature B; *P* = 0.041, MZ B; *P* = 0.0085, PerC: aPtC B; *P* = 0.054, aPC B; *P* = 0.051. For T cell, CD4^+^ T cells in the spleen and mLN analysis (BALB/c and C57BL/6; n = 8 each, + / + ; n = 10, + /–; n = 8, –/–; n = 10). CD8^+^ T cells (BALB/c and C57BL/6; n = 5 each, + / + ; n = 7, + /–; n = 6, –/–; n = 6). For + / + vs –/–: Spl: CD4^+^T; *P* = 0.0139, CD8^+^T; *P* = 0.047. (**C**) 2 mo V_H_11KI.Pla2g2a + / + , + /–, –/– mice analyzed for aPtC B cells in B cells and CD4/CD8 T cells (n = 3 each) and aPtC B cells in PBL B cells. For + / + vs –/–: PBL aPtC; *P* = 0.0377. (**D**) 2 mo ATAμκTg.Pla2g2a + / + , + /–, –/– mice were analyzed for ATA B, ATA^–^ B, and IgM^b^ B cells in B cells (n = 3 each).

Foetal HSCs are Lin28^+^Let7^–^ and an increasing Lin28^–^ Let7^+^ HSC population occurs between 3–4 weeks after birth in mice^58^. At the neonatal-3 wk stage, the frequencies of Th2-type ILC2s and NKT2 cells are higher than those in adults, and B-1-derived B1a cell generation is higher, followed by MZ B cell generation starting at approximately the 3-wk stage^16,59–61^. In contrast to the predominant FO B cells in adults that are T-cell dependent for Ig secretion, B1a and MZ B cells are able to secrete Igs without T cell help^62,63^. To assess whether loss of Pla2g2a in the BALB/c background leads to changes in B and T cell developmental outcomes, including B1a cells, from the neonatal to 2 mo adult stages, we analyzed BALB/c mice. Pla2g2a + / + , + /–, –/– mice. At the 3-wk stage, although total B cells (CD19^+^) were unchanged in the spleen and mLN, Pla2g2a^-/-^ BALB/c mice showed consistently lower B1a cells in the mLN, and a lower frequency of B1a cells in the spleen was found in approximately 60% (3/5) of these mice (Fig. 3A, left). For T cells, Pla2g2a^-/-^ BALB/c mice showed a slight increase in total T cells (CD3^+^) with lower CD4^+^ T cells versus increased CD8^+^ T cells in the spleen, and this lower CD4/CD8 ratio was also trending in mLN (Fig. 3A, right).

At the 2 mo adult stage, the frequencies of immature stage (as T1 and T2) splenic B cells were higher in Pla2g2a^-/-^ mice and MZ B cells were lower. B1a cells remained slightly lower in the spleen (Fig. 3B left). Total B1a cells in the PerC were similar between the BALB/c Pla2g2a^+/+^ and Pla2g2a^-/-^ mice, which differed from the lower frequencies of B1a cells in the PerC of C57BL/6 mice at 2 mo of age Fig. 3B. However, originally generated TdT-independent B1a cells, such as aPtC B cells (V_H_11/V_k_9)^64^ and anti-phosphocholine (aPC) B cells (PC-BSA/kappa^+^, confirmed V_H_107/V_k_22)^65^, were reduced at the adult stage in the Pla2g2a^-/-^ mice (Fig. 3B, right). The IgH sequencing of B1a cells confirmed the change in IgHs and reduced V_H_11^+^ and V_H_12^+^ aPtC B cells, and some Pla2g2a^–/–^ mice showed increased J558 IgH, such as V1-55 IgH B1a cells as often found in C57BL/6 B1a cells (Fig. S1A,B)^66,67^. Analysis of T cells at 2 mo of age continued to show a lower CD4/CD8 ratio in the spleen and mLNs of Pla2g2a^-/-^ mice, consistent with a further lower CD4/CD8 ratio in C57BL/6 mice Fig. 3B.

To confirm the lower frequency of early-generated aPtC and ATA B1a cells in adult Pla2g2a^-/-^ mice, we analyzed V_H_11KI and ATAμκTg mice crossed with Pla2g2a^–/–^ mice. As shown in Fig. 3C, although aPtC cells were predominantly PerC B1a in Pla2g2a + / + , + /–, –/– mice, lower frequencies of aPtC B cells were observed in Spl, mLN, PP in intestine, and LN in the Pla2g2a^–/–^ mice, and circulating aPtC cells were also lower in PBL. ATAμκTg mice also showed lower numbers of ATA B cells in the Spl, mLN, PP, and LN, and lower numbers in the PerC in the Pla2g2a^–/–^ background Fig. 3D. As originally found in the C57BL/6 background Fig. 1E, ATA-negative B cells, including endogenous IgM^b^ cells, were increased in Pla2g2a^–/–^ mice Fig. 3D. Although the PBLs of C57BL/6 mice also exhibited a lower frequency of B1a cells and a lower CD4/CD8 T cell ratio, the CD4/CD8 T cell ratio in PBLs was unchanged in the Pla2g2a^+/+^ vs. Pla2g2a^–/–^BALB/c mice at 2–12 mo of age (Fig. S2). Thus, although not completely identical between C57BL/6 and BALB/c.Pla2g2a^-/-^ mice, low original B1a BCR cells increased with low IL-5 and decreased the CD4/CD8 ratio in Pla2g2a^-/-^BALB/c mice, as C57BL/6 mice confirmed that Pla2g2a deficiency substantially impacts BALB/c mice to mimic the phenotype of C57BL/6 mice.

### Changed NKT cells and increased MAIT cells in Pla2g2a^–/–^ mice, and the loss of CD1d alter the alters the levels of B1a and MZ B cells

**Figure 4:**
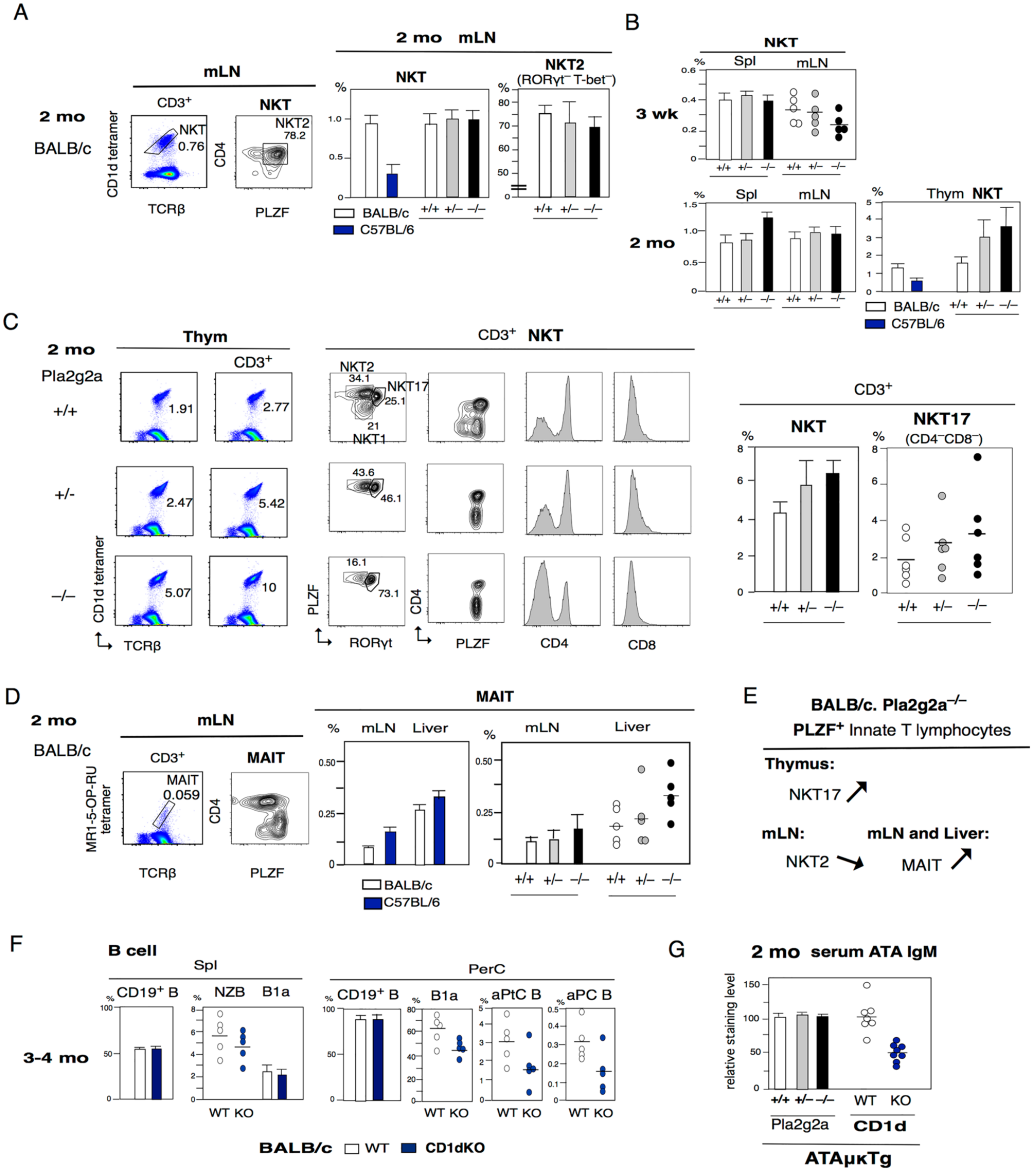
In PLZF^+^ T cells, there were increased NKT17 cells in the thymus and increased MAIT cells in the mLN and liver of Pla2g2a^–/–^ mice. Loss of CD1d alters B1a, and MZ B cells. The majority of data showed the mean ± SE, and some showed individual data with mean percentages and + / + vs –/– *P*-value. (**A**) 2 mo mLN NKT and NKT2 cells in Pla2g2a + / + , + /–. –/– (NKT n = 6 each, NKT2 n = 4 each; mean ± SE). NKT cells in BALB/c and C57BL/6 mice (n = 4 each; mean ± SE). (**B**) 3-wk and 2-mo stages of NKT cells present in spl and mLN CD3 + T cells in Pla2g2a + / + , + /–, –/– mice (n = 5 each). In 3 wk Pla2g2a^–/–^ mice in mLN, low NKT cells; for + / + vs –/–, *P* = 0.069. Analysis of NKT cells in Pla2g2a + / + , + /–. –/– mice (n = 6 each; mean ± SE), and in BALB/c and C57BL/6 (n = 4 each; mean ± SE). (**C**) Left: Thymus NKT cells in total thymus versus CD3^+^ T cells, and CD3^+^ NKT cell analysis for PLZF^hi^ RORγt^–^ (T-bet^–^) NKT2, PLZF^med^RORγt^+^ NKT17, and PLZF^lo^RORγt^-^ (T-bet^+^) NKT1, and PLZF, CD4, CD8, in Pla2g2a + / + , + /–, –/– mice. Right; NKT and PLZF^med^CD4^-^CD8^-^ RORγt^+^ NKT7 cells in CD3^+^ T cells (n = 6 each). For + / + vs –/–: NKT17 *P* = 0.269. High NKT and NKT17 cells in Pla2g2a^-/-^ mice. (**D**) MAIT analysis in mLN and liver in Pla2g2a + / + , + /–, –/– mice (n = 5 each). For + / + vs –/–: Liver MAIT *P* = 0.068. Increased MAIT in Pla2g2a^-/-^ mice, similar to higher C57BL/6 than BALB/c mice (n = 2 each). (**E**) In Pla2g2a^–/–^ mice, the PLZF^+^ T cell type changed; high NKT17, low NKT2, and increased MAIT cells. (**F**) 3–4 mo B cell analysis in spl and PerC in BALB/c WT and BALB/c.CD1dKO mice (n = 5 each). Spl MZB; *P* = 0.020, PerC: B1a; *P* = 0.076, aPtC B;*P* = 0.0418, aPC B; *P* = 0.139. (**G**) 2-mo serum ATA IgM levels analyzed under ATAμκTg.Pla2g2a + / + . + /–. –/– (+ / + ; n = 10, + /–; n = 20, –/–; n = 9) backgrounds were similar. In contrast, ATA IgM levels were lower in ATAμκTg.CD1dKO mice than ATAμκTg mice (n = 6 each), *P* = 0.0003.

In Fig. 2E, we found that CD3^+^ T cells in the mLN of 2-mo-old Pla2g2a^-/-^ mice had low IL-5 levels. The total frequency of CD1d-reactive NKT cells was not reduced, in contrast to the substantially lower NKT cells in C57BL/6 mice Fig. 4A. However, PLZF^+^CD4^+^ NKT2 cells (RORγt^–^ T-bet^–^) were lower in Pla2g2a^-/-^ mice Fig. 4A. At the early 3-wk stage, total NKT cells were consistently lower in mLNs (mostly PLZF^+^CD4^+^) in Pla2g2a^–/–^ mice Fig. 4B, together with lower B1a cells in mLNs Fig. 3A. In the spleen, a lower frequency of NKT cells was found at 3 wk in 60% (3/5) of Pla2g2a^-/-^ mice. Then, at the 2 mo adult stage, increased total NKT cells were observed in the spleen Fig. 4B. In the thymus of these 2 mo mice, BALB/c.Pla2g2a^–/–^ mice were often showed more strongly increased NKT cells than Pla2g2a + / + mice, in compared with lower C57BL/6 mice (Fig. 4B right). This increase in thymic NKT cells in BALB/c.Pla2g2a^–/–^ mice were associated with increased PLZF^+^ RORγt^+^ CD4^–^CD8^–^ NKT17 cells Fig. 4C. Mucosal-associated invariant T cells (MAIT) recognize MR1 on hemopoietic cell types and are generated by many strains of bacteria and yeast. MAIT cells also arise from PLZF^+^ origin, and TCRβ^+^MR1^+^ MAIT cell analysis in intestinal-mLN and in liver showed dominantly Th1 and Th17 cells, and exhibit higher frequency in C57BL/6 than BALB/c mice as known^68^. BALB/c Pla2g2a^-/-^ mice with higher NKT17 cells in the thymus also showed consistently higher MAIT in the mLN and liver Fig. 4D. In conclusion, innate PLZF^+^ origin T cells in Pla2g2a^-/-^ BALB/c background mice have increased bacteria-reactive NKT17 cells and MAIT cells, but decreased NKT2 cells Fig. 4E.

Since B cells are CD1d^+^ and react with NKT cells^69^, we analyzed the loss of CD1d in BALB/c mice (CD1dKO) to consider the role of NKT2 cells on these Pla2g2a–/– effects. NKT cells promote B cell proliferation and antibody production and control^70^. Three- to four month-old CD1dKO mice showed consistently lower MZ B cells in the spleen and lower total B1a cells in the PerC, with decreased aPtC and aPC B1a cells Fig. 4F. Thus, together with ILC2s, Th2-type NKT2 cells originally contributed to the B1a cell increase from the neonatal to adult stage, including in intestine-mLNs with IL-5. As previously known, the increased total NKT cells in NKT Tg mice showed a slight decrease in sensitive CD1d^high^ MZ B cells^71^, increased total NKT cells in the spleen in 2-mo-old Pla2g2a^-/-^ mice Fig. 4B showed decreased MZ B cells Fig. 3B, and MZ B cells also decreased in CD1dKO mice Fig. 4F. In ATAμκTg.CB17 mice at the 2 mo stage, ATA B cells were low in the Pla2g2a^–/–^ background in a variety of lymphoid tissues Fig. 3D. However, the serum ATA IgM levels in PBLs were similar between BALB/_C_ mice Pla2g2a + / + , + /–, –/– mice at the 2 mo stage Fig. 4G, which differed from the low serum ATA IgM in C57BL/6 mice ATAμκTg mice Fig. 1F. However, ATAμκTg.CD1dKO mice showed strongly low serum ATA IgM levels Fig. 4G. This suggests that low serum ATA IgM in C57BL/6 PBL resulted from lack of Pla2g2a, and further lower total NKT cell generation with low NKT2 cells and not increased NKT17 cells is due to different SLAM expression by unique C57BL/6 mice.

### Continued connection between NKT2 and ATA B cells and impact of the loss of CD1d on ATA B cell tumor development

**Figure 5:**
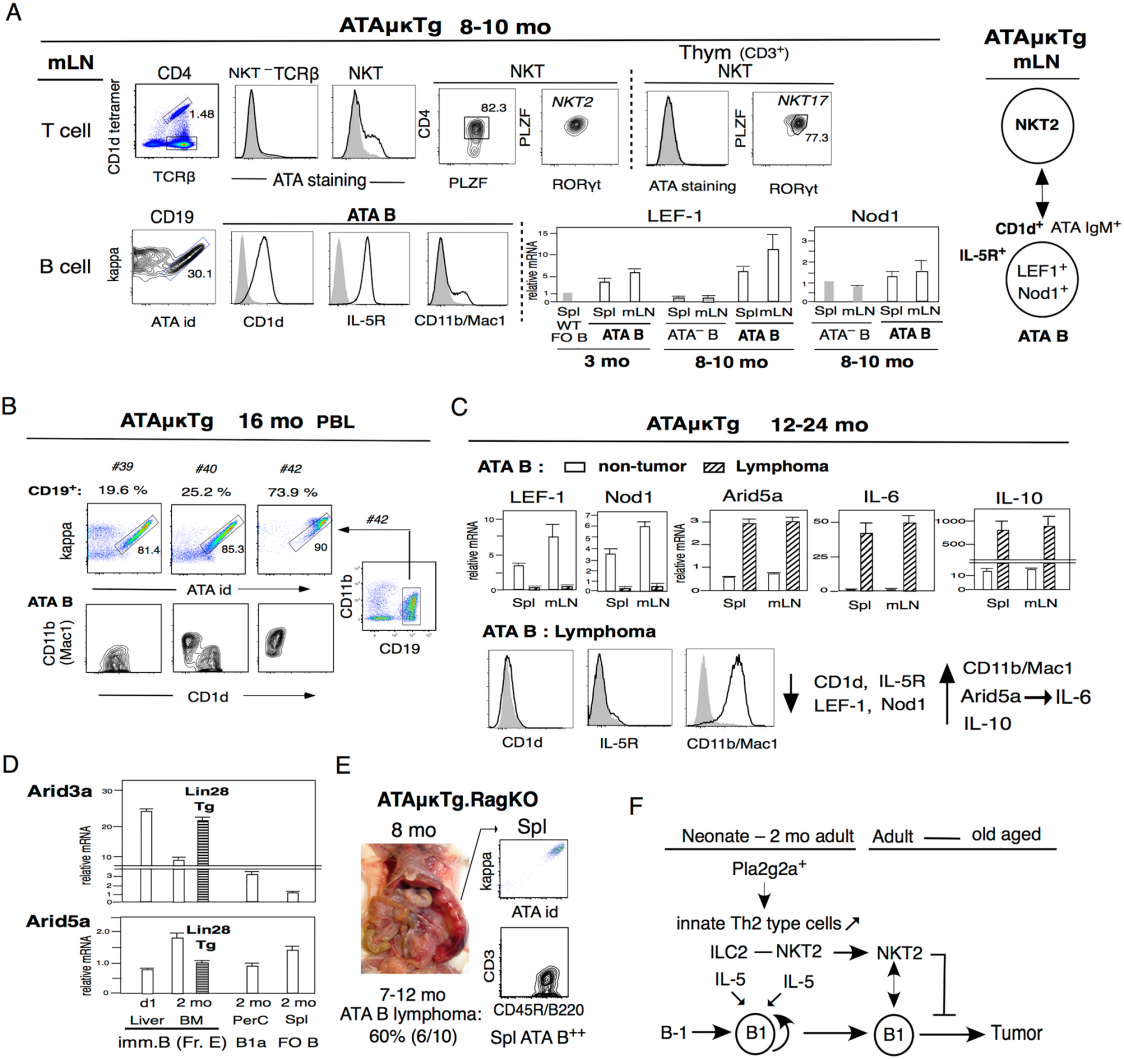
NKT2 binding to CD1d^+^ATA B cells and the loss of CD1d in ATA B cells induce tumors in old aged individuals. (**A**) NKT cell and ATA B cell analysis in mLN in 8–10 mo ATAμκTg C.B17 mice. Top: NKT cells in the mLN were predominantly PLZF^+^CD4^+^RORγt^low^ NKT2 cells compared with increased NKT17^+^ cells in the thymus at 8–10 mo. Bottom: ATA B cells in mLNs with CD1d^+^, IL-5R^+^, and low CD11b/Mac1, thus, NKT2 cells in mLNs have the ability to bind to ATA IgM. LEF-1 and Nod1 qRT-PCR: FO B in Spl versus ATA B cells in Spl and mLN for LEF-1 at 3 mo (n = 3 each; mean ± SE), and ATA B versus ATA^–^ B cells in Spl and mLN for LEF-1 and Nod1 at 8–10 mo (n = 4 for ATA B, n = 3 for ATA^–^ B; mean ± SE). (**B**) 16 mo ATA B cell analysis in PBL B cells with CD1d and CD11b/Mac1 analysis. (**C**) Twelve to 24 mo ATA B cells with nontumor versus tumor stage in spl and mLN. LEF-1, Nod1, Arid5a, IL-6, and IL-10 qRT-PCR (n = 4 each; mean ± SE). ATA B lymphoma was all CD1d^-^, IL-5R^-^, and high CD11b/Mac-1. (**D**) 2 mo adult BM Fr. E (immature B) stage Arid3a and Arid5a mRNAs compared with Lin28^+^Tg (n = 3 each; mean ± SE). (**E**) RagKO ATAμκTg mice showed 60% (6/10) generation of ATA B lymphoma at 7–12 mo with high Spl lymphoma without CD11b/Mac1 increase. (**F**) Pla2g2a^+^ promotes increased IL-5 production from innate Th2-type cells (ILC2s and NKT2s) with B1a cell growth from neonates to adults and then generates NKT2 control B1 cells.

In adult humans, IL-5-secreting NKT cells, known as NKT2, critically regulate anti-blood group A-PBC B1a secretion in collaboration with B1a cells^72^. In Pla2g2a^+^ATAμκTg in C.B17 mice at the 8–10 mo stage, NKT cells were still present in mLNs, predominantly NKT2 cells, in contrast to increased NKT17 cells in the aging thymus with microbiota changes Fig. 5A. ATA B cells secrete ATA IgM continuously (also IgM switches to IgA in old age^10^). Although increased NKT17 cells in the thymus were unable to bind ATA IgM, NKT2 cells in mLNs showed some binding to ATA IgM, but not NKT^–^ T cells Fig. 5A. ATA B cells in mLN were continuously CD1d^+^ and IL-5R^+^, and CD11b (Mac-1) was slightly increased at the 8–10 mo stage. Thus, NKT2 cells can bind to CD1d^+^ ATA BCR-expressing B cells and IL-5-IL-5R can continuously promote IgM (also IgA) secretion. As found in LEF-1^+^ (lymphoid enhancer binding factor-1) human CLL^73^, LEF-1 mRNA was slightly higher in ATA B cells than normal FO B cells in the spleen and mLN at the 3 mo adult stage, and increased in 8–10 mo aged mice, particularly in mLN Fig. 5A. Nod1, which recognizes the products of intestinal commensal bacteria, is constantly higher in B1a cells from the neonatal stage to aged mice^74^, and was also higher in mLN Fig. 5A.

During further aging, as in 16-mo-old mice, there were different stages of ATA B cells in PBLs. As shown in Fig. 5B, normal ATA B cells in PBL were continuously CD1d^+^ and CD11b/Mac1^low/–^ as in mouse #30, and mouse #40 showed both CD1d^+^CD11b^–^ and CD1d^–^ CD11b^+^ ATA B cells. Mouse #42 showed predominantly CD19^+^ B cells in PBL with CD1d^–^ CD11b^+^ATA B cells. This #42 mouse developed CLL in PBL that became strong Spl and mLN lymphoma. When ATA B cells with lymphoma versus nonlymphoma were analyzed at the 12–24 mo stage Fig. 5C, we found that the high ATA B lymphomas lacked both CD1d and IL-5R expression and had increased CD11b/Mac1, as previously shown^10^. LEF-1 expression became negative, and Nod1 was also decreased. IL-6 expression was negative in nonlymphoma ATA B cells. ATA B CLL/lymphoma cells were IL-6^+^, and IL-6-binding Arid5a^75^ was also increased. IL-10 was detected at the nonlymphoma ATA B cell stage, and the lymphoma stage showed strongly increased IL-10 expression and CD1b/Mac1^+^IL-6^+^IL-10^++^ in ATA B lymphoma.

Increased Arid3a^+^ immature B cells (Fr. E) in the neonatal stage allows B1a cell generation and the presence of IL-5 is important for the increase in B1a cells, which do not require IL-6^76^. In contrast, Arid5a is higher at the Fr. E stage in adult BM than in neonatal liver and this is the outcome of Lin28^–^ adult HSCs, since Lin28 Tg decreased Arid5a Fig. 5D. Arid5a is higher in FO B cells than in B1a cells. Loss of CD1d by B1 cells leads to loss of NKT2 contraction of B1, which then changes to IL-6^+^ B1 B cells with tumor generation in old age Fig. 5C. When the protein coding gene Rag1 (recombination activating 1) was lost in ATAμκTg mice, resulting in the presence of IL-5-producing ILC2s and MPP^type2^ but lack of NKT2 cells as negative mature T cells in RagKO mice, ATA B cells strongly increased and lymphoma occurred at an early 7–12 mo stage (60%, 6/10), mostly in the spleen Fig. 5E. From these data, we conclude that after IL-5^+^, B1a cells increase at the neonatal-young adult stage, and NKT2 cells continuously control initially increased B1 cell growth. The loss of CD1d in old age results in tumor development Fig. 5F.

## Discussion

The majority of mammals express the Pla2g2a protein, including in humans. Analysis of Pla2g2a^–/–^ BALB/c mice confirmed that after birth, the presence of Pla2g2a is important to control microbiota diversity, allowing increased development of Th2-type ILC2 and NKT2 cells, CD4^+^ T cells, and B1a cells. Previously, it was found that BALB/c d14 fetal liver hematopoietic cell transfer to C57BL/6 fetus generated NKT1 cells, but not NKT2 cells, similar to C57BL/6^77^. Additionally, introduction of the NZB mouse SLAMF6 (Ly108) gene into C57BL/6 mice resulted in increased NKT2 cell generation in NKT cells, but total NKT cell levels were not as strongly increased as lower total NKT cells in normal C57BL/6 mice^78^. These data led us to further confirm that the presence of Pla2g2a is critical for higher ILC2 and NKT2 cells in the neonatal stage and IL-5-dependent initial B1a cell increase. Distinctly low NKT2 cells occurred in Pla2g2a-negative mouse background C57BL/6 mice.

IL-5-producing Th2-type ILC2 cytokine generation is IL-25- and/or IL-33-dependent, and the production of IL-5 was most likely reduced as a result of altered intestinal bacteria in the absence of Pla2g2a. The gastrointestinal tract hosts a diverse community of microbes including bacteria, fungi, helminths, and viruses. Among T cells with NKT cells, Th1 cells react to infection with intracellular bacteria or viruses, and Th17 cells contribute to immunity against extracellular bacteria and fungi. In contrast, Th2 cells are induced by helminths, and play a central role in mediating allergic disorders and asthma upon commensal-dependent expression^79^. Bacteria controlling Pla2g2a are expressed in Paneth cells and secreted into goblet cells and mucus in the intestine, and the enzyme degrades bacterial membranes^80,81^. Secreted Pla2g2a also binds to the M-type receptor PLA2R1 expressed on intestinal stem cells and plays an important role as an intestinal stem cell factor contributing to homeostasis, inflammation, and cancer^28,81^. Thus, bacteria controlling Pla2g2a allow IL-25 and/or IL-33 expression to generate ILC2 cells and increase Th2-type NKT2 cells. In the fetal/neonatal stage, Lin28 expression in HSCs leads to loss of Let7 expression. For T cells, it is known that Let7 loss allows PLZF^+^ and Myc^+^ expression, leading to Bach2^low^ cells which are primed for Th2-type cytokine secretion^49^. Lin28^**+**^ HSCs are also miR150^–^, which allows the expression of Myb, and Myb also increases the development of GATA3^+^–Th-POK^+^ CD4^+^ T cells^82^. Thus, in most normal mice, PLZF + NKT2 cells and B1a cells are predominantly generated in the neonatal-young stage as an outcome from Lin28^+^Let7^–^ HSCs together with Pla2g2a^+^, as opposed to in adults.

Pla2g2a-negative C57BL/6 mice showed more reduced frequencies of Th2-type ILC2s and NKT2, CD4^+^ T, and B1a cells than BALB/c.Pla2g2a^-/-^ mice. Additionally, C57BL/6 mice showed low total NKT cells, including at the adult stage, which is different from BALB/c mice. Pla2g2a^-/-^ mice. We speculate that the additional difference of in SLAM expression in C57BL/6 mice further altered the generation of these cell types together with Pla2g2a loss. SLAM family genes (located on mouse 1qH3) encode immune cell-specific receptors and the SLAM family is distinct in C57BL/6 mice as haplotype 1, compared with the majority of mice that are considered haplotype 2^24^. Several members of the SLAM family are expressed in the initial differentiation from lymphoid stem cells (including in the HSC-MPP stage) and participate in the development of both the T and B cell lineages^83^. At the neonatal stage, SLAMF1 and SLAMF6 (Ly108) are important for NKT cell generation, and Ly108 increases PLZF expression with Th2 cytokine production by PLZF^hi^NKT2 cells^84,85^. In contrast to the majority of mice, C57BL/6 mice have several SLAM family receptor differences with a lower tyrosine phosphorylation signal leading to low total NKT cell and NKT2 cell generation and altered lymphocyte activation. Thus, the lack of Pla2g2a production together with different SLAM family receptor expression in C57BL/6 mice contributes to the subtle differences with BALB/c mice.Pla2g2a^-/-^ mice.

In Pla2g2a^-/-^ mice, the CD4/CD8 ratio is reduced. CD4^+^ T cells are decreased in the early 3-wk stage and continue to be reduced through the adult 2-mo stage in Pla2g2a^-/-^ mice. The CD4/CD8 ratio difference was more pronounced in the spleen and mLNs than in the thymus, indicating that this difference is not only due to the initial T cell-generating stage. In BALB/c Pla2g2a^–/–^ mice, T cells generated from the thymus were more markedly affected in the spleen and mLNs, but not in PBLs. C57BL/6 mice have a comparatively low CD4/CD8 T cell ratio, including in the spleen, mLNs, LNs, and PBLs (Fig. S2)^20^. Previously, several analyses of CD4/CD8 ratio differences showed that high B7-1 (CD80) and B7-2 (CD86) levels leads to reduced CD4/CD8 T cell ratio, and a lack of CD80 and CD86 results in an increased CD4/8 T cell ratio^86^. Both high expression of CD80 and CD86 in a model of pulmonary inflammation contributed to the development of airway hyperresponsiveness (AHR)^87^, and CD86 is rapidly upregulated during an immune response^88^. In the intestine, AHR is expressed by intraepithelial lymphocytes (IELs), Th17 cells, and ILC3 cells, and AHR ligands are found in food, intestinal microbiota, and environmental contaminants^89^. Several bacterial taxa also affect the differentiation of native T cells. It was found that when CD4^+^ T cells in IELs were stimulated by *lactobacillus reuteri,* a common Firmicutes in the intestine, AHR was activated, which downregulated CD4^+^ generating ThPOK, leading to CD4^+^CD8aa^+^ DP T cells in the IEL^90^. These data indicate that bacterial increases in Pla2g2a^–/–^ mice impact the immune response and can change the CD4/CD8 ratio.

IL-25 is expressed in the gut, lung, thymus, and central nervous system^91^. In the gastrointestinal tract, tuft cells are the major IL-25 source to stimulate ILC2s, and tuft cells in the medulla area of the thymus are also critical for the initial development and intrathymic function of NKT2 cells. IL-33 also generates ILC2 cells, and IL-33 is expressed by a wide variety of cell types, including epithelial barrier tissues and lymphoid organs^47^. In the intestine, fibroblasts with systemic infection secrete IL-33 and react with intestinal epithelial differentiation, including IL-33 receptor (ST2)-expressing stem cells and Paneth cells. In addition to IL-5, IL-13 secreted from ILC2s in response to IL-25 and/or IL-33 expression stimulates mucus production by goblet cells and also binds to tuft cells^41,92^. In goblet cells, Muc2 cells are present, and anti-goblet cell (GC) B cells including anti-Muc2 are generated in MZ B cells in the spleen by transport of Muc2 with dendritic cells from intestine to spleen in BALB/c and C.B17 mice, whereas a-GC B cells are strongly low/negative in C57BL/6 mice among MZ B cells^60^. Lower a-GC BCR expression in BALB/c mice Pla2g2a^–/–^ MZ B cells as compared to Pla2g2a^+/+^ mice were found in the presence of ATAμκ Tg (Fig. S1C). Thus, IL-25/IL-33-stimulated ILC2 cells produce IL-13, which impacts the intestine, including goblet cells, affecting the early generation of MZ B cells. Thus, decreased Th2-type ILC2 cells in Pla2g2a^–/–^ mice can also change initial MZ B cell generation.

NKT cells are stimulated by CD1d which is expressed by a variety of cells, including intestinal epithelia^93^. CD1d-deficient mice were found to have an altered bacterial composition^94^. In the intestine, invariant natural killer T (iNKT) cells, as the dominant NKT cells, are present among IELs and within the lamina propria (LP) and bind to the CD1d-expressing Paneth cells^95,96^ to affect Pla2g2a secretion. Reduced NKT cells were observed in the mLNs of 3-wk-old BALB/c mice.Pla2g2a^-/-^ mice. On the other hand, adult BALB/c.Pla2g2a^-/-^ thymus showed increased NKT cells with increased NKT17 cells in Pla2g2a^–/–^ mice, distinct from SLAM different NKT low C57BL/6 mice. The reduction in NKT cells in Pla2g2a^–/–^ mice at the 3 wk stage was the result of the initial reduction of the original neonatally-generated NKT2 cells. Changing IL-25/IL-33 expression to IL-17/ IL-22 expression in Pla2g2a^–/–^ mice from Th17 type cells may have increased continuously in response to higher bacterial reactivity. MAIT cells are dependent on bacteria and absent in germ-free mice^97^, and MALT cells have the capacity to produce IL-17 and IFNγ, similar to Th17 and Th1 cells^68^. We found that MALT cells increased under the Pla2g2a^-/-^ background. These 3-wk and 2-month analyses all confirmed reduced neonatal-generated Th2-type cells and increased Th17-type cells in the bacterial increase in BALB/c mice.Pla2g2a^–/–^ mice.

In adults, the majority of B cells are derived from Lin28^–^ B-2 development. However, surviving innate autoreactive and polyreactive B1 B cells contribute immediate innate responses in infection through Ig secretion and cytokines before the T-cell dependent B2 B cell response. Then, in aging 8–15 mo old mice, some PerC deposited B1 B cells and tissue presenting B1 B cells can more effectively migrate and increase in PBL. At the nontumor stage, neonate-generated ATA B cells and NKT2 cells can interact together, and NKT2 cells continue to control B1 B cells through IL-5-IL-5R signaling to promote B1 B cell self-renewal with IgM secretion, as also found in humans^72^. Then, upon further old aging, approximately half of the ATAμκ Tg mice strongly increased B1 B cells in PBL and developed leukemia/lymphoma in spl and mLN under CD1d reduction. Human CLL B cells are often originally CD1d^++^, but CD1d reduction occurs in CLL with increased intracellular protein and altered adhesion with reduced survival^98,99^, and the downregulation of CD1d occurs during some infections, such as viruses, including HIV (human immunodeficiency virus)^100,101^. RagKO.ATAμκ Tg mice lack mature T cells, including NKT/NKT2 cells, but still have IL-5-producing ILC2s, and these mice showed earlier and strongly increased ATA B cell tumor generation. Thus, we conclude that NKT2 cells are involved in controlling the B1 B cell increase that leads to initial CLL development.

When CD1d deficiency abrogated NKT2-B1a cell interactions and B1 B cell lymphoma strongly increased in these animals, one reason may be the *Cdkn2a* gene difference in the BALB/c background, which has the potential to strongly promote B1 B cell growth^12^. However, we also considered a potential role for IL-6. In mouse CD1d^–^ ATA B cell lymphoma, we observed reduced LEF-1 and increased IL-6 and Arid5a. In humans, before the CLL stage, MBL cells in PBL are LEF^+^^73^, and at the CLL stage, cells become Wnt3^+^ and/or LEF^+^ and further increase to LEF^++^ leads to poor prognosis with reduced CD1d and decreased survival^99,102,103^. Strongly increased MCL (mantle cell lymphoma) from CLL is caused by LEF-1^–^, and LEF^–^ also decreases the survival of CLL. IL-6 expression is also found in human CLL^104^. The strong ATA B cell lymphoma stages in mice were CD1d^–^, LEF-1^–^, and IL-6^+^ with increased Arid5a. IL-6 is a pleiotropic cytokine with diverse expression^105^, and the original neonatal-generated B1a cells and nonlymphoma ATA B1 cells were IL-6^–^. At the early Lin28^+^/Let7^–^ stage, both IL-5 and IL-6 increase can occur^106^. In contrast to IL-5, the increase in B1a cells does not require IL-6, but instead a lack of IL-6 (IL-6^–^) increases the frequency of B1a cells and decreases B2 B cells^76,107^. IL-6 is continuously available in adults at various expression levels, and IL-6 increases Th17 T cells along with TGFβ ^108^. At the adult stage, Arid5a is increased in the immature B cell stage, while the neonatal stage is lower as shown here. Arid5a binds to IL-6 mRNA to promote IL-6 production. It is known that high IL-6 is found in inflammatory diseases and cancers, including in B cell tumors^109,110^. Thus, we propose that Arid5a-IL-6 interactions in CD1d^–^ (IL-5R^–^) B1 B cells may have promoted the development of lymphoma, different from NKT2 connecting CD1d^+^B1 B cells.

Under normal conditions, Pla2g2a modulates insulin sensitivity and metabolism and the regulation of whole-body metabolism^111^. However, strongly increased levels of secreted Pla2g2a can lead to inflammatory diseases, such as arthritis (rheumatoid), sepsis, and cardiovascular disease in humans^27^, and Pla2g2a^–^ C57BL/6 mice are less susceptible to such diseases. Secreted Pla2g2a can bind to the M-type receptor (PLA2R1) in mice, and cPLA_2_ – Wnt signaling can occur, including in the intestine^81^. This Pla2g2a binding can control and also promote susceptibility to cancer. Secreted Pla2g2a binds to macrophages (Mø) to induce the production of IL-6^112^. Inflamed Møs (such as when stimulated with LPS or IL-1β) increase Arid5a^+^ to produce IL-6^75^, and IL-6 increases Pla2g2a secretion in the lung and liver^113^. Thus, Pla2g2a can be challenged in old aged pathologies. Correspondingly Pla2g2a^+^ BALB/c and C.B17 mice showed increased B1a cells in neonatal adults and certain BCR B1 B cells generated CLL/lymphoma in old age as found in unmutated CLL in humans. Although various changes occur in old aged individuals, the sufficient presence of B1 B cells is important in adults to provide immediate protection against bacteria and viral and control autoantigens. The Pla2g2a^+^ background is required at the beginning of the B1a cell increase in the neonatal to adult stage.

## Conclusion

In summary, our study confirmed that the presence of bacteria controlling Pla2g2a is important for immunity to allow neonatal to adult stage and old aged outcome. The presence of Pla2g2a allowed increased IL-5 production from Th2-type ILC2s and NKT2 cells to allow the generation of a B1a cell increase from the neonatal to adult stage. Generated initial NKT2 cells continuously control initially increased B1 cell growth.

## Methods

### Mice

BALB/cAnN, C.B17, and C57BL/6J mice are housed in the Fox Chase Cancer Center.

(FCCC) Laboratory Animal Facility, which is accrediated by the Association for Assessments and Accreditation of Laboratory Animal Care International. V_H_3609/V_k_21-5 ATAμκ Tg mice (3369)^114^, V_H_Q52 VDJ aMyIIA knock-in mice (ON25)^7^, V_H_11 VDJ aPtc knock-in mice (VH11t)^115^, Lin28b Tg (Rag2-Lin28b-λ5 LCR Tg) mice and Arid3a Tg (Rag2-Arid3a-Poly Tg) mice^1^ were originally generated in the C.B17 background and described in each previous paper as listed here. All these Tg^+^ and KI background C.B17 mice were maintained, crossed with C57BL/6 J mice (> 8 generations) and maintained. Eμ-hTCL1 Tg mice were originally C57BL/6 and crossed into the C.B17 background, and Thy-1^–^ cells were generated in C.B17 and C57BL/6 backgrounds as previously described^6^. Arid3a knockout mice were on the C57BL/6 background, and Rag1 KO mice were on the BALB/c background. Pla2g2a knockout and CD1d knockout mice on a BALB/c background were originally obtained from Jackson Laboratory. BALB/c and C.B17 background ATAμκ Tg, V_H_11 KI, and aMyIIAKI mice were first crossed with Pla2g2a knockout BALB/c mice, generating Pla2g2a heterozygous mice, then bred to obtain Pla2g2a + / + , + /–, –/– littermates for comparison, and Pla2g2a + / + , + /–, –/– were also compared using similar aged stages. Additionally, CD1d KO and Rag1 KO mice were crossed with ATAμκ Tg.C.B17 mice.

### Flow cytometry analysis, and ELISA immunoassay

Cell preparation for immunofluorescence staining, multicolor flow cytometry analysis, sorting, and monoclonal antibody reagents has been described previously^7,10^. Further antibodies used here for analysis are as follows: IL-7Ra (SB/199), IL-2Ra (7D4), PLZF (9E12), RORγt (B2D), T-bet (4B10), TCRβ (H57-597), IL-5Ra (DIH37), CD1d (1B1), and PC (PC-BSA with kappa). Anti-ATA B V_H_3609/V_k_21-5 (19A4), anti-PtC V_H_11id (3H7) with anti-V_k_9id (13B5), and anti-aMyIIA B V_H_Q52/V_k_9 (24E1) have been described previously in original papers. The CD1d tetramer and MR1-5-OP-PU tetramer were obtained from the NIH tetramer core facility. For flow cytometry Pla2g2a + / + , + /–, –/– B and T cell analysis, CD19^+^ for total B cells and immature B (AA4^+^), MZ B (CD21^hi^CD23^lo/–^), B1a (B220^lo^CD5^+^), aPtC B, aPC B, ATA B cell percentage in CD19^+^ cells, CD3^+^ for total T cells and CD4^+^ T, CD8^+^ T cell percentage in CD3^+^ cells, for Pla2g2a + / + , + /–, –/– mouse comparison. For NKT (NKT1, NKT2, NKT17) and MAIT cell analysis, PE CD1d tetramer or PE MR1-5-OP-PU tetramer was bound to samples for 30 min at first, followed by CD3, CD4, CD8, CD19, TCRβ, PLZF, RORγt, and T-bet analysis. For ELISA IL-5 analysis, sorted CD3^-^CD19^-^ cells, CD3^+^ T cells, and CD19^+^ B cells (as an IL-5^–^ control) from PP and mLN cells (using 3 mice each for Pla2g2a + / + , + /–, –/– to sort) were stimulated with IL-2 + IL-25 and IL-2 + IL-33 (10 ng/ml each)(from Biolegend), and 4 days later, IL-5 ELISA analysis was performed by coating with 5 μg/ml TRFK5 (anti-IL-5), followed by detecting with biotin TRFK4 (1/100) (anti-IL-5), followed by alkaline phosphatase-conjugated avidin (AP Av). IL-5 (Biolegend) was used as a control for the IL-5 ratio.

### B-lineage sorting from liver and BM

For the sorting of B-lineages from neonatal liver (1d) and adult BM (2 mo) for qRT-PCR analysis, selection markers of early B-lineage cells (CD19^+^B220^+^AA4^+^, CD11b/Mac1^–^Gr1^–^, LybC^–^Ter119^–^CD3^–^) together with CD43^–^IgM^–^CD24^hi^ Pre-B (Fr. D), and IgM^+^IgD^–^ immature B (Fr. E) were used as described previously^1^.

### qRT-PCR and RT-PCR assays

Gene expression was quantitated by real-time PCR, using TaqMan assays for 35 cycles from Applied Biosystems, an ABI 7500 real-time thermal cycler, and ABI software (Life Technologies). Relative gene expression levels were normalized as a ratio using β-actin values for mRNA as a standard for quantitation (to calculate relative mRNA levels). For gut PP and mLN IL-5 qRT-PCR, unstimulated Pla2g2a^+/+^ mouse data (-) were used as mRNA1. The Bach2 RT-PCR primer set was as follows:

5’-CAGTGAGTCGTGTCCTGTGC-3’ and 5’-TTCCTGGGAAGGTCTGTGAT-3’.

TaqMan Assay primer sets for Lef1 (Mm00550265_m1), Nod1/Card4 (Mm00805062_m1), Arid5a (Mm00524454_m1), IL6 (Mm00446190_m1), and IL10 (Mm00524454_m1) were purchased from ThermoFisher (catalog #4331182).

### B cell leukemia/lymphoma diagnosis

PBL analysis was performed at 2 mo and every 3 months for ATAμκTg with Pla2g2a + / + , + /–, –/–. In ATAμκTg mice (Pla2g2a^+^), the mice showing a predominance of ATA^+^ B1 B cells (CD19^+^B220^lo^CD5^+^ B1a, or CD19^+^B220^lo^CD5^lo/–^ of B1a origin) in PBL were subjected to tissue analysis in comparison to the lack of a strong increase in ATA^+^ B1 B cells at a similar old age. Mostly high ATA B1 B cells in PBL were associated with splenomegaly and increased in mLN with B1 B cell dominance similar to ATA B lymphoma. Rag1KO.ATAμκTg mice were subjected to PBL analysis every 2–3 months, followed by tissue analysis which showed an early increase of in spleen cells, also often liver increases, and mostly not mLN increases.

### Statistical analyses

Statistics were calculated using a t-test on Excel.

### Ethics statement

All mouse studies were approved by the FCCC Institutional Animal Care and Use Committee (IACUC) under protocol number 87–17. All animal euthanasia was performed in accordance with the American Veterinary Medical Association Guidelines for the Euthanasis of Animals and all animal studies were performed according to the ARRIVE Guidelines.

## Supplementary Information

Supplementary Information 1.

Supplementary Information 2.

Supplementary Information 3.

## Data Availability

All data are available from the corresponding author upon reasonable request.
