# Supplementary material for: Pla2g2a promotes innate Th2-type immunity lymphocytes to increase B1a cells
Source: Sci Rep. 2022 Sep 1;12:14899. doi: 10.1038/s41598-022-18876-4 (PMC9437038; doi:10.1038/s41598-022-18876-4)
Supplement: Supplementary file 1 — Supplementary Information 1. [file 41598_2022_18876_MOESM1_ESM.pdf]

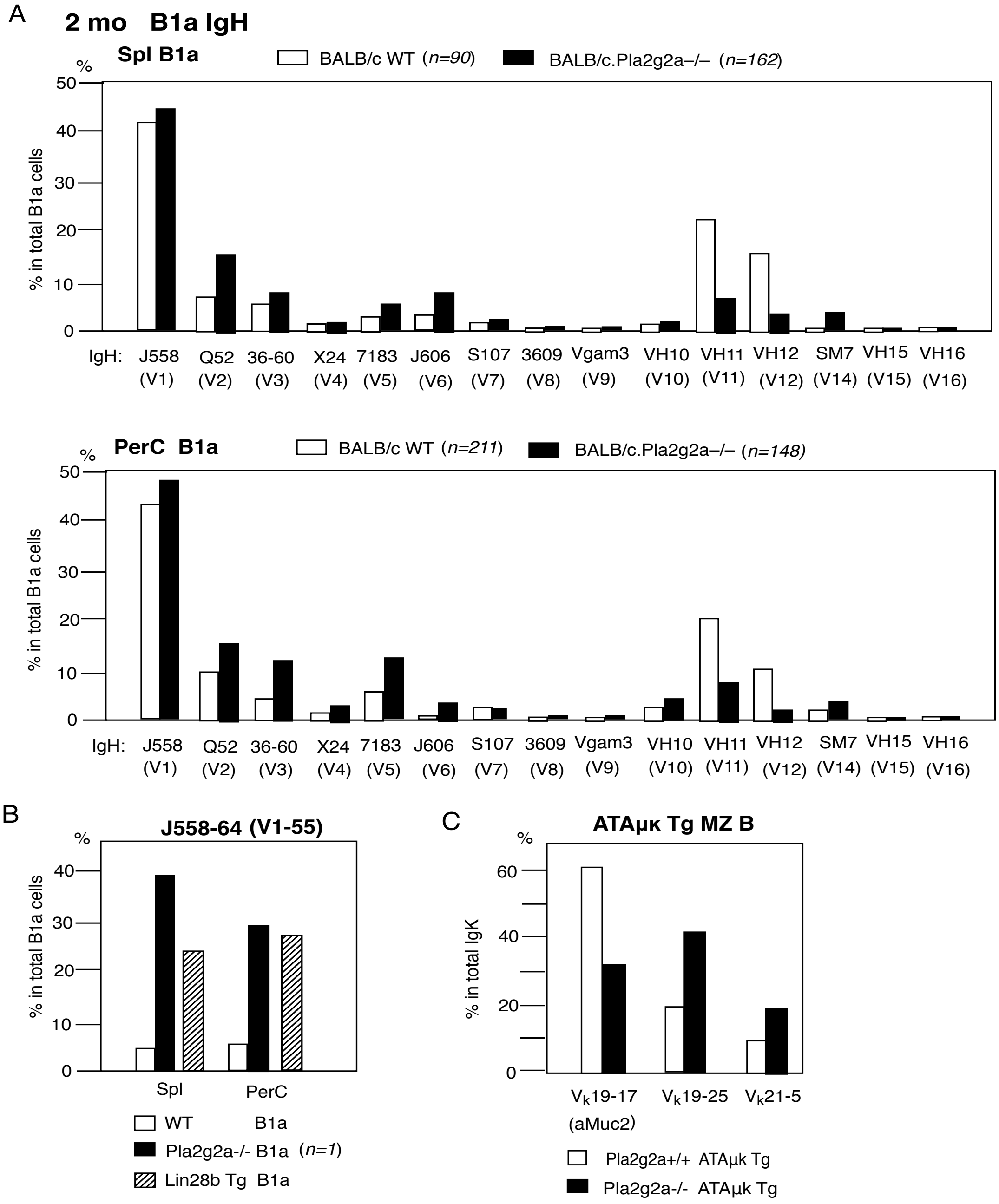

Figure S1. B1a IgH and MZ B IgK gene analysis of 2 mo old Pla2g2a$^{-/-}$ mice. (A) 2 mo B1a cell V_H genes in spleen and PerC in BALB/c WT and BALB/c Pla2g2a$^{-/-}$ mice. The IgV_H gene family name list was originally generated (Hayakawa, J.Exp.Med 2016; 213:3007) and is available in IMGT (www.imgt.org). (B) V_H J558-64 is often found in C57BL/6 B1a cells in early stage and 2 mo, and also TCL1 Tg$^+$ B1 tumor (Yang, eLIFE 2015; 4:e09083)(Holodick, Front Immunol 2016; 7:108)(Hayakawa, J.Exp. Med 2016; 213:3007). One 2 mo old Pla2g2a$^{-/-}$ mouse showed high J558-64, which was also often found in Lin28b Tg$^+$ BALB/c B1a cells (Hayakawa, Front Immunol 2019;10:457)). (C) MZ B cells in ATAμκTg mice showed changed IgK, often V_k19-17 as aGC/aMuc2, and V_k19-25 and V_k21-5 (Ichikawa, J.Immunol. 2015;194:606), while Pla2g2a$^{-/-}$ mice showed lower V_k19-17.
