# Supplementary material for: Pla2g2a promotes innate Th2-type immunity lymphocytes to increase B1a cells
Source: Sci Rep. 2022 Sep 1;12:14899. doi: 10.1038/s41598-022-18876-4 (PMC9437038; doi:10.1038/s41598-022-18876-4)
Supplement: Supplementary file 2 — Supplementary Information 2. [file 41598_2022_18876_MOESM2_ESM.pdf]

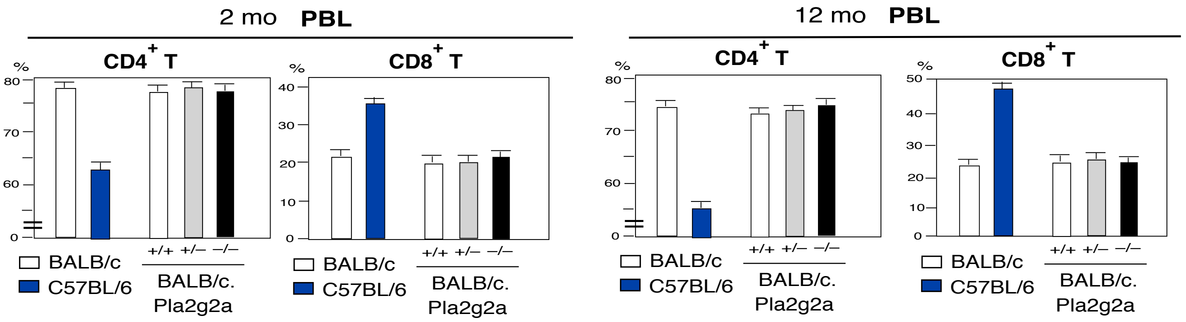


**Figure S2 Similar CD4$^+$ and CD8$^+$ T cells in BALB/c.Pla2g2a+/+, +/–, –/– PBL, in contrast to lower CD4$^+$ T and higher CD8$^+$ T cells in C57BL/6 than BALB/c mice.** (A) PBL from 2 mo old Pla2g2a+/+, +/–, –/– mice; n=10 each. (B) PBL from 12 mo old Pla2g2a+/+; n=9, Pla2g2a+/–; n=7, Pla2g2a–/–; n=5. 2 mo and 12 mo old BALB/c and C57BL/6 mice; n=3 each
