# Supplementary material for: Pla2g2a promotes innate Th2-type immunity lymphocytes to increase B1a cells
Source: Sci Rep. 2022 Sep 1;12:14899. doi: 10.1038/s41598-022-18876-4 (PMC9437038; doi:10.1038/s41598-022-18876-4)
Supplement: Supplementary file 3 — Supplementary Information 3. [file 41598_2022_18876_MOESM3_ESM.pdf]

Supplemental experimental procedures for Fig. 2A

Quantitative RT-PCR and Western blotting

For B cell subset purification for qRT-PCR (mRNA) and Western blot, neonatal liver (1d) and adult BM (2 mo) Pre-B (Fr.D; $CD43^{-}IgM^{-}CD24^{hi}$) and immature B (Fr. E; $IgM^{+}IgD^{-}$) cells were purified from early B-lineage cells ($CD19^{+}B220^{+}AA4.1^{+}$, $CD11b/Mac1^{-}Gr1^{-}$, $LybC^{-}Ter119^{-}$ $CD3^{-}$) in C.B17 mice. For adult spl immature B ($AA4.1^{+}$), MZ B ($CD21^{hi}CD23^{lo/-}$), and spl/PerC B1a ($B220^{lo}CD5^{+}$) were purified from $CD19^{+}$ cells. For Western blot, these B cell subsets purified by cell sorting (2 x 10^{6} cells per tube), and cell lysates were subjected to SDS-PAGE and immunoblotted with anti-Bach2 and anti-β actin (Bethyl Laboratories).

Bach2:
Forward: CAGTGAGTCGTGTCCTGTGC
Reverse: TTCCTGGGAAGGTCTGTGAT

Original blots

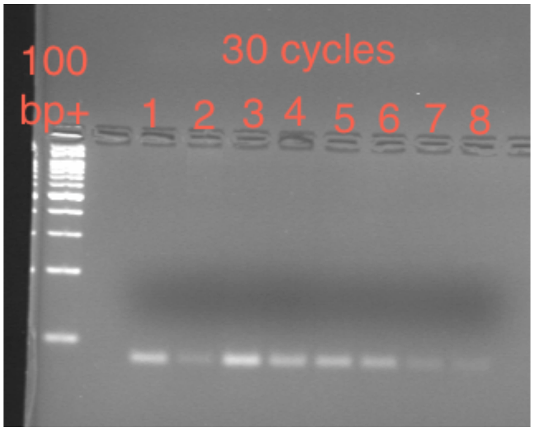

Bach2

— 100 bp

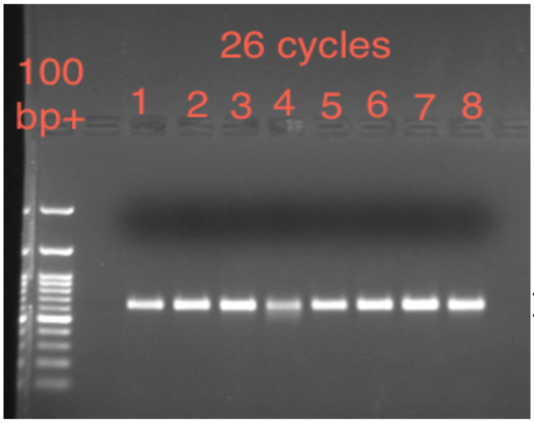

β-actin

— 676 bp
— 517 bp

1d BM
1: pre B
2: imm B

2mo BM
3: pre B
4: imm B

Spl
5: imm B
6: Fo B
7: B1a

PerC
8: B1a

Arid3a knockout mice and Arid3a transgenic mice

Arid3a knockout mice B6N(Cg)-Arid3a$^{tm1b(KOMP)wtsi}$ with C57BL/6 (B6) background were originally purchased from the Jackson Laboratory, then generated C.B17 background. Arid3a transgenic (Rag2-Arid3a-PolyA Tg) mice line was originally we generated in the C.B17 background. For Arid3a Tg mice generation [1], we used a mouse recombination activating gene 2 (Rag2) promoter element consisting of about 7.5 kb fragment upstream of the Rag2 coding region, and a surrogate light chain locus control region, to generate a Rag2-Lin28b-λ5LCR construct. 1d Liver and 2 mo BM Imm B (Fr. E) comparison with WT mice (C.B17) by qRT-PCR.

Lin28b Tg (Rag2-Lin28b-λ5 LCR)

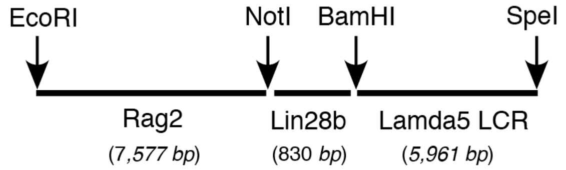

1. Hayakawa K, Li YS, Shinton SA, Bandi SR, Formica AM, Brill-Dashoff J, Hardy RR: Crucial Role of Increased Arid3a at the Pre-B and Immature B Cell Stages for B1a Cell Generation. Front Immunol. 2019;10:457
